# Nutrition, Nutraceuticals and Bioactive Compounds in the Prevention and Fight against Inflammation

**DOI:** 10.3390/nu15112629

**Published:** 2023-06-05

**Authors:** Stanisław Surma, Amirhossein Sahebkar, Maciej Banach

**Affiliations:** 1 Faculty of Medical Sciences in Katowice, Medical University of Silesia, Medyków 18, 40-752 Katowice, Poland; surma.stanislaw96@gmail.com; 2 Polish Lipid Association (PoLA), Sterlinga 27/29/205, 90-212 Lodz, Poland; 3 Biotechnology Research Center, Pharmaceutical Technology Institute, Mashhad University of Medical Sciences, Mashhad 9177948954, Iran; amir_saheb2000@yahoo.com; 4 Applied Biomedical Research Center, Mashhad University of Medical Sciences, Mashhad 9177948954, Iran; 5 Department of Biotechnology, School of Pharmacy, Mashhad University of Medical Sciences, Mashhad 9177948954, Iran; 6 Department of Preventive Cardiology and Lipidology, Medical University of Lodz (MUL), Rzgowska 281/289, 93-338 Lodz, Poland; 7 Cardiovascular Research Centre, University of Zielona Gora, Zyty 28, 65-417 Zielona Gora, Poland; 8 Department of Cardiology and Congenital Diseases of Adults, Polish Mother’s Memorial Hospital Research Institute (PMMHRI), Rzgowska 281/289, 93-338 Lodz, Poland; 9 Ciccarone Center for the Prevention of Cardiovascular Disease, Johns Hopkins University School of Medicine, 600 N. Wolfe St, Carnegie 565-G, Baltimore, MD 21287, USA

Chronic low-grade systemic inflammation is a key factor involved in the pathogenesis of many diseases and their complications (Figure 1) [1,2,3].

SARS-CoV-2—severe acute respiratory syndrome coronavirus 2; MetS—metabolic syndrome; DMT2—diabetes mellitus type 2; MAFLD—metabolic-associated fatty liver disease; ASCVD—atherosclerotic cardiovascular disease; CKD—chronic kidney disease.

Chronic low-grade systemic inflammation is characterized by several important factors: (1) the damage-associated molecular pattern (DAMP; “exposome”, metabolic dysfunction, and tissue damage) is the causative factor; (2) the duration is persistent and non-resolving; (3) it is low-grade inflammation; (4) outcomes include collateral damage; (5) age is also a crucial factor; and (6) it is silent, with no canonical standard biomarkers [1]. Inflammation is closely related to oxidative stress. Reactive oxygen species (free radicals; ROS) are produced as a result of the stimulation of DAMP Toll-like receptors (TLRs). As a result, there is an increase in the production of pro-inflammatory cytokines, including interleukins (IL) 1β, 6, and 18, and the development of inflammation [4].

Due to all these factors, controlling inflammation is crucial, both before and after the onset of the disease [1,2,3,5].

Inflammation plays a special role in the pathophysiology of atherosclerotic cardiovascular disease (ASCVD) [6]. ASCVD is the leading cause of death worldwide. In 2021, 4.75 (95% CI: 3.76–5.58) million people worldwide died from ASCVD [7], and these numbers might be even higher in 2023 due to the COVID-19 pandemic and long-COVID complications [8,9].

In a meta-analysis of 14 studies by Li et al., including 83,995 participants, it was shown that higher levels of hs-CRP were associated with a significantly higher risk of all-cause mortality (RR = 1.75; 95%: 1.55–1.98), cancer mortality (RR = 1.25; 95% CI: 1.13–1.38), and CV mortality (RR = 2.03; 95% CI: 1.65–2.50) [10]. A meta-analysis of 54 prospective studies conducted by Kaptoge et al. showed that elevated CRP concentrations significantly increased the risk of ASCVD, independently of other conventional risk factors [11]. A meta-analysis of 22 studies conducted by Ni et al. showed that moderately elevated and high CRP concentrations significantly increased the risk of all-cause mortality (RR = 1.30; 95% CI: 1.20–1.41 and RR = 1.75; 95% CI: 1.59–1.92, respectively) and cardiovascular (CV) mortality (RR = 1.43; 95% CI: 1.22–1.68 and RR = 2.02; 95% CI: 1.70–2.41, respectively). High CRP concentration was also associated with a higher risk of cancer mortality (RR = 1.32; 95% CI: 1.21–1.45) [12].

A significant role of chronic low-grade systemic inflammation in ASCVD was demonstrated in the Canakinumab Anti-Inflammatory Thrombosis Outcome Study (CANTOS) involving 10,061 patients with acute coronary syndrome (ACS) and an elevated level of the high-sensitivity C-reactive protein (hsCRP) concentration (≥2 mg/L). Patients were administered canakinumab (an anti-IL 1β monoclonal antibody) for 3 months at a dose of 50, 150, or 300 mg. The authors showed that canakinumab at the dose of 150 mg significantly reduced the risk of nonfatal stroke, nonfatal ACS, and CV mortality (HR = 0.85; 95% CI: 0.74–0.98), in addition to reducing the risk of hospitalization for unstable coronary artery disease (CAD), which led to urgent revascularization (HR = 0.83; 95% CI: 0.73–0.95) [13]. However, at the same time, canakinumab significantly increased the risk of fatal sepsis, and therefore it is not an appropriate treatment for CVD patients. Among other anti-inflammatory drugs, colchicine has been shown to reduce CV risk in many available trials. A meta-analysis of 11 randomized controlled trials (RCTs) by Bytyçi et al. showed that colchicine significantly reduced the risk of major CV, stroke, CV hospitalization in patients with CAD, and recurrent ACS [14]. The recent results of the CLEAR-Outcomes trial also confirmed the role of bempedoic acid in the significant reduction in CRP concentrations, especially in patients with increased concentrations at baseline (>2 mg/L). The mechanism responsible for this is associated with the inhibition of the 5′AMP-activated protein kinase, but further analysis is still needed on whether this effect might be associated with the reduction in CVD outcomes [15].

Taken together, these findings indicate that canakinumab and colchicine significantly reduce the CV risk, thus confirming the significant contribution of chronic low-grade systemic inflammation in the pathogenesis of these diseases.

The influence of lipid-lowering drugs on chronic low-grade systemic inflammation is also significant. Treatment of lipid disorders (mainly through a reduction in low-density lipoprotein cholesterol (LDL-C)) is the most preventive measure in the context of ASCVD risk [16,17]. Statins are the most important drugs in the treatment of lipid disorders [17,18]. In a meta-analysis of 37 RCTs by Zhang et al., including 17,410 patients with lipid disorders or CAD, statin use was associated with a significant decrease in CRP (WMD = −0.97; 95% CI: −1.31 to −0.64), and the most favorable long-term effect in this respect was observed using atorvastatin at a dose of 80 mg/day [19]. In a meta-analysis of 26 studies by Kandelouei et al., it was also shown that the use of statins decreased the concentration of CRP and hs-CRP [20]. In their study involving 3745 ACS patients, Ridker et al. revealed that low CRP concentrations have better clinical outcomes after treatment with statins despite the concentration of LDL-C [21]. In their other study involving 17,802 patients without lipid disorders and with increased hs-CRP concentration, they showed that rosuvastatin decreased hs-CRP concentrations by 37%, leading to a 44% reduction in serious CV events (HR = 0.56; 95% CI: 0.46–0.69) [22].

It should be emphasized that persistently increased hs-CRP, regardless of the effective reduction in atherogenic lipoproteins, is still observed in 30–40% of all statin trials’ participants. If an even more aggressive LDL-C cutoff of 55 mg/dL is considered, then 50% of patients might still have elevated levels of hs-CRP [23]. Moreover, ezetimibe used as monotherapy did not affect CRP concentrations, and if even a slight reduction is observed in some available meta-analyses, this effect is indirect (e.g., via a reduction in lipoprotein(a); Lp(a)) and not clinically significant [24,25]. However, as Ballantyne et al. revealed in a study involving 628 patients with hypercholesterolemia, the combination of ezetimibe with atorvastatin significantly reduced CRP levels compared with atorvastatin monotherapy [26]. Similarly to ezetimibe, proprotein convertase subtilisin/kexin type 9 inhibitors (PCSK9i) did not significantly affect the hs-CRP concentration, as shown in a meta-analysis of 16 RCTs (2546 patients) by Sahebkar et al. (WMD = 0.002 mg L^−1^, 95% CI: −0.017 to 0.021) [27]. They might, however, contribute to the reduction in vascular wall inflammation via direct and indirect effects [28].

Apart from lipid-lowering effects, bempedoic acid, as already mentioned above, also exerts anti-inflammatory effects via the activation of the AMP-activated protein kinase pathway in immune cells. Banach et al. confirmed this finding in a pooled analysis of data from phase-III RCTs involving 3623 patients with hypercholesterolemia [29]. This is in agreement with another meta-analysis of 10 RCTs by Cicero et al., which showed that bempedoic acid reduced hs-CRP levels by 27.03% (95% CI: −31.42 to −22.64) [30].

Based on the data of 6136 high-risk patients from the REGARDS study (Reasons for Geographical Additionally, Racial Differences in Stroke), low hs-CRP levels (<2 mg/L) were associated with reduced risk of incident CHD, incident stroke, and CHD death, whereas low LDL-C levels (<70 mg/dL) were not associated with protective effects, which supports other data concerning the importance of inflammatory processes in the pathogenesis of CVD [31]. A meta-analysis of nine studies by Berkley and Ferro showed that the reduction in CRP concentration in patients at high residual inflammatory risk led to a significant reduction in the risk of ASCVD despite non-elevated lipid concentrations or the use of lipid-lowering therapy [32].

In summary, despite its high effectiveness in CV prevention, lipid-lowering treatment may not be effective enough in reducing CRP concentrations.

Given that chronic low-grade systemic inflammation is an important residual risk factor of ASCVD (and many other diseases—Figure 1) and that the anti-inflammatory effects of available therapies are not sufficient, various natural ingredients in the diet may play a role in supporting this therapy [3]. Many natural food ingredients, eating patterns, and nutraceuticals can influence chronic low-grade systemic inflammation (Figure 2) [3,33].

Currently, the topic of natural products and their different applications is very popular and much debated. Various studies have been performed that highlight different effects of nutraceuticals and/or food supplements in patients with different ASCVD risk scores. Unfortunately, most of these data are derived from small cohort studies, and the conclusions do not provide clear answers on the real benefits and applications of nutraceuticals. Therefore, in a modified presentation of the recommendations from the International Lipid Expert Panel (ILEP), we summarized data on nutraceuticals with proven anti-inflammatory properties (Table 1) [34].

In conclusion, a limited number of nutraceuticals have significant anti-inflammatory properties. Thus, research is underway to expand knowledge on the impact of food ingredients, diets, and nutraceuticals on the inflammatory process.

In this Special Issue of *Nutrients* entitled “Nutrition, Nutraceuticals and Bioactive Compounds in the Prevention and Fight against Inflammation”, several articles have been published [35,36,37,38,39,40,41,42,43,44,45,46], which significantly contribute to the knowledge in this area.

For some, despite their favorable anti-inflammatory effects, data are still insufficient for inclusion in the recommendations. In a study by Shirvani et al., 24 men undergoing the army combat readiness test (ACRT) were randomized to supplementation with 500 mg of *Origanum vulgare* or a placebo. It was shown that the use of oregano was associated with an increase in oxidative capacity [35]. The results of this study indicate that *Origanum vulgare* exhibits antioxidant activity, resulting in an anti-inflammatory effect. Experimental studies have revealed that the anti-inflammatory properties of carvacrol (a polyphenol found in oregano) are dose-dependent. Excessively high doses of carvacrol may increase oxidative stress, inflammation, and apoptosis [36]. A novel, functional miso-type sauce based on legumes also exhibited antioxidant activity, which was demonstrated in a clinical study by Papagianni et al. [37].

Another significant nutraceutical with a proven anti-inflammatory effect is curcumin [47]. Mahmoudi et al. performed a bioinformatic analysis, highlighting the impact of curcumin on several critical liver cirrhosis genes mainly involved in extracellular matrix communication, focal adhesion, and the response to oxidative stress, which may slow down the process of liver cirrhosis [38]. In another bioinformatic analysis, Mahmoudi et al. also showed that curcumin may improve or inhibit the progression of metabolic-associated fatty liver disease (MAFLD) through the activation/inhibition of MAFLD-related genes [39]. Another analysis by the same group revealed that curcumin targeted several important genes involved in diabetes pathogenesis [40]. Berberine is also a beneficial nutraceutical in the prevention and treatment of MAFLD, as it improves lipid and glucose metabolism, energy homeostasis, and gut microbiota [41]. In the context of diabetes, the results of a randomized clinical trial by Butler et al. are important, which showed no improvement in glycemic indices during supplementation of 60 g daily date fruit or raisins, though neither had a deleterious effect on glycemic control over 12 weeks, indicating their safety when consumed in type 2 diabetes [42].

The influence of vitamin D on cardiovascular diseases has recently been extensively studied and discussed [48]. In the study by Moin et al., vitamin D was not found to have potential anti-inflammatory properties, and in a non-obese population with polycystic ovary syndrome (PCOS) matched for age and insulin resistance, circulating inflammatory proteins and matrix metalloproteinases were not elevated and did not correlate with 25(OH)D_3_, or its epimer 3epi25(OH)D or 1,25(OH)_2_D_3_ [43].

From an epidemiological point of view, the results of a mouse study by Lee et al. are also of interest, in which the impact of Citrus junos Tanaka (a citrus fruit that contains bioactive flavonoids including naringin) on lung damage caused by PM10 particulate matter was assessed. It was shown that pro-inflammatory cytokines and NF-κB/apoptosis signaling-related markers were decreased in the intervention group compared with the control [44]. A diet rich in flavanols (e.g., quercetin) improves the function of the vascular endothelium and has lipid-lowering and antihypertensive effects in various groups of patients with CVD [45]. The same positive anti-inflammatory effects were also observed for the lychee extract, especially in the form of a flavanol-rich lychee fruit extract (FRLFE) [49]. Coffee and tea (particularly green) rich in polyphenols also have anti-inflammatory and cardioprotective effects in patients with various CVDs [50,51]. The use of natural products may also be useful in supporting the treatment of patients with inflammatory bowel disease [46].

Inflammation is related to nutritional status; hence, the prognostic inflammatory and nutritional index (PINI) is a valuable diagnostic tool, in which CRP and α1-acid glycoprotein as well as albumin and transthyretin are assessed. The PINI aims to evaluate the nutritional and inflammatory status of patients presenting inflammatory syndromes with or without denutrition [52].

Based on the above studies, we strongly believe that dietary interventions based on natural products are crucial to reduce systemic and/or regional low-grade inflammation and thus improve general health, reduce the risk of chronic diseases, delay and/or prevent cardiovascular diseases, and improve prognosis. Given the significance of this research topic, we would like to thank and congratulate all the authors who submitted their papers for consideration in this Special Issue, and we encourage the editors and authors of *Nutrients* to continue the discussion and contribute to further advances in this field.

## Figures and Tables

**Figure 1:**
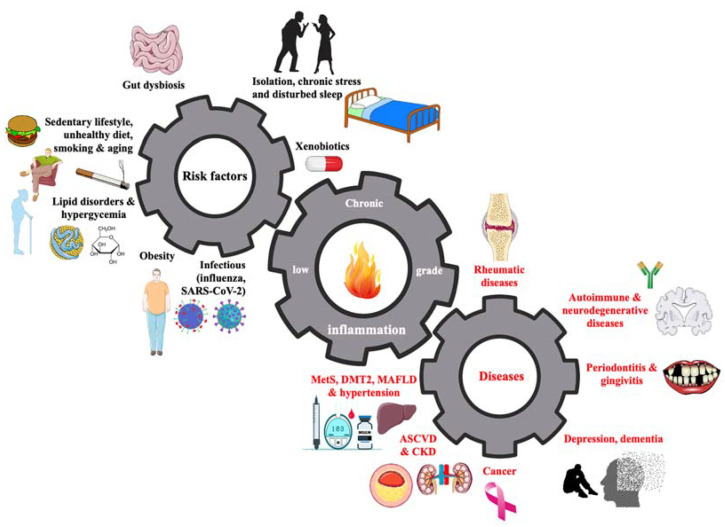
Causes and consequences of low-grade systemic chronic inflammation.

**Figure 2:**
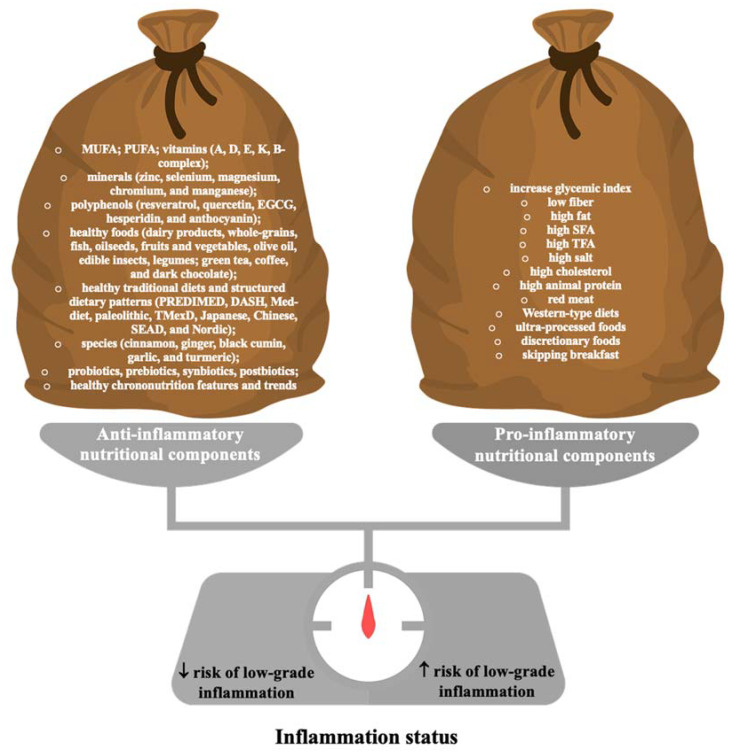
The influence of various dietary components, eating habits, and nutraceuticals on inflammatory status in humans. MUFA—monounsaturated fatty acids; PUFA—polyunsaturated fatty acids; EGCG—epigallocatechin-3-gallate; PREDIMED—Prevention of Dieta Mediterranea; DASH—Dietary Approaches to Stop Hypertension; TMexD—traditional Mexican diet; Med-diet—Mediterranean diet; SEAD—Southern European Atlantic Diet; SFA—saturated fatty acids; TFA—trans fatty acids.

**Table 1:** International Lipid Expert Panel (ILEP) recommendations on the effect of nutraceuticals on inflammatory parameters. Modified based on [34]. * Increasing the risk of atrial fibrillation. The colors symbolically represent the class and the level of evidence.

Nutraceuticals	Class of Evidence	Level of Evidence
Omega-3 fatty acids *	**I**	**A**
Red yeast rice	**I**	**A**
Flavonoids	**IIa**	**B**
Soy	**IIa**	**A**
Curcumin	**IIa**	**B**
Omega-6 fatty acids	**IIb**	**B**
Berberine	**IIb**	**B**
Garlic	**IIb**	**B**
Bergamot	**III**	**C**
Lupin	**III**	**C**

Legend: class of evidence: 1—is recommended/is indicated; IIa—should be considered; IIb—may be considered; III—is not recommended; level of evidence: A—data derived from multiple randomized clinical trials or their meta-analyses; B—data derived from a single randomized clinical trial or large non-randomized studies; C—data from preclinical or in vitro studies.
